# Ghost roads and the destruction of Asia-Pacific tropical forests

**DOI:** 10.1038/s41586-024-07303-5

**Published:** 2024-04-10

**Authors:** Jayden E. Engert, Mason J. Campbell, Joshua E. Cinner, Yoko Ishida, Sean Sloan, Jatna Supriatna, Mohammed Alamgir, Jaime Cislowski, William F. Laurance

**Affiliations:** 1 Centre for Tropical Environmental and Sustainability Science, and College of Science and Engineering, James Cook University, Cairns, Queensland Australia; 2 College of Arts, Society and Education, James Cook University, Townsville, Queensland Australia; 3 Research Center for Climate Change, and Department of Biology, University of Indonesia, Depok, Indonesia; 4 Present Address: Thriving Oceans Research Hub, School of Geosciences, University of Sydney, Camperdown, New South Wales Australia; 5 Present Address: Department of Geography, Vancouver Island University, Nanaimo, British Columbia Canada

**Keywords:** Environmental sciences, Environmental impact, Ecology

## Abstract

Roads are expanding at the fastest pace in human history. This is the case especially in biodiversity-rich tropical nations, where roads can result in forest loss and fragmentation, wildfires, illicit land invasions and negative societal effects^1–5^. Many roads are being constructed illegally or informally and do not appear on any existing road map^6–10^; the toll of such ‘ghost roads’ on ecosystems is poorly understood. Here we use around 7,000 h of effort by trained volunteers to map ghost roads across the tropical Asia-Pacific region, sampling 1.42 million plots, each 1 km^2^ in area. Our intensive sampling revealed a total of 1.37 million km of roads in our plots—from 3.0 to 6.6 times more roads than were found in leading datasets of roads globally. Across our study area, road building almost always preceded local forest loss, and road density was by far the strongest correlate^11^ of deforestation out of 38 potential biophysical and socioeconomic covariates. The relationship between road density and forest loss was nonlinear, with deforestation peaking soon after roads penetrate a landscape and then declining as roads multiply and remaining accessible forests largely disappear. Notably, after controlling for lower road density inside protected areas, we found that protected areas had only modest additional effects on preventing forest loss, implying that their most vital conservation function is limiting roads and road-related environmental disruption. Collectively, our findings suggest that burgeoning, poorly studied ghost roads are among the gravest of all direct threats to tropical forests.

## Abstract (web summary)

An effort to map roads in the Asia-Pacific region finds that there are 3.0–6.6 times more roads than other sources suggest, and that unmapped ‘ghost roads’ are a major contributor to tropical forest loss.

## Main

By the middle of this century, Earth is expected to have some 25 million km of new paved roads relative to 2010—enough to encircle the planet more than 600 times^1^. Roads serve a number of important societal functions, such as promoting trade and increasing access to natural resources and arable land^7,8,12^. Without effective planning and law enforcement, however, roads can also unleash a Pandora’s box of environmental ills and societal challenges^2,13–16^. Unfortunately, many new roads are being constructed informally or illegally, especially in lower-income nations where governance is often hindered by corruption and ineffective law enforcement^7,15^. These ‘ghost roads’, invisible on official road maps, are one of the most vexing direct threats to tropical forests and their wild and human inhabitants^6,7^.

We define ghost roads operationally as those missing from the two leading global-road datasets: the Global Roads Inventory Project^17^ (GRIP) and OpenStreetMap^18^ (OSM). Ghost roads include informally or illicitly constructed roads, bulldozed tracks in logged forests, roads in palm-oil plantations and other roads that are missing from existing road datasets for various reasons. Such roads can be either paved or unpaved, although most are unpaved. Ghost roads are being constructed by a range of people, including legal or illegal agriculturalists, miners, loggers, land grabbers, land speculators and drug traffickers, among others^6–9^.

The accuracy and completeness of existing road maps vary greatly among nations and regions, and are typically poorest in developing nations with large forest estates^19,20^. To assess the extent of ghost roads, we carried out an intensive sampling effort (1.42 million plots of 1 km^2^ each) across a range of human-altered and native-forested regions of Borneo, Sumatra and New Guinea—three of the world’s largest continental islands. We manually mapped and digitized roads on each island using recent (circa 2019) high-resolution satellite imagery in Google Earth. Mapping was conducted by 210 trained volunteers or researchers whose individual mapping accuracy was quality-checked by one or more co-authors of this study, using test datasets (Supplementary Information and Supplementary Fig. 1). Each mapper was required to attain an accuracy of more than 90% on test datasets (including road omissions and commissions) before commencing road-mapping.

After generating high-accuracy road data, we (1) compared the extent of roads from our data directly with those from the two leading global-road datasets (GRIP^17^ and OSM^18^); (2) assessed how roads and other key socioeconomic and environmental variables influence forest loss; (3) gauged how protected areas affect the proliferation of roads and associated environmental disruption; and (4) used a temporal analysis to assess whether roads tend to precede, or follow, deforestation across our study area.

## Road extent and density

**Fig. 1:**
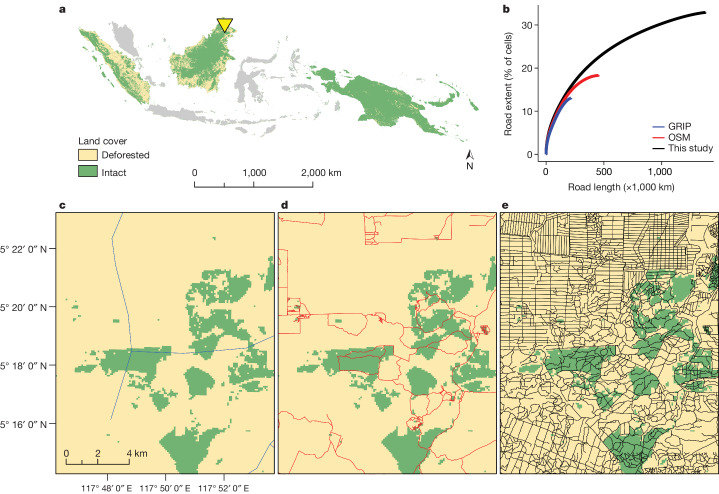
Road density in the tropical Asia-Pacific region is much higher than indicated by available global datasets. **a**, The study region, comprising part or all of Indonesia, Malaysia and Papua New Guinea (the yellow triangle shows the location of inset panels **c**–**e**). **b**, Cumulative plots comparing the total length of roads and proportion of land potentially affected by roads (road extent, percentage of 1-km^2^ cells containing roads) in this study versus data from OSM and GRIP. Sites are ordered from highest to lowest road length. **c**–**e**, Mapped roads in a landscape in Sabah, Malaysian Borneo, as shown by GRIP (**c**; blue lines, imagery circa 2018), OSM (**d**; red lines, circa 2020) and this study (**e**; black lines, circa 2019), respectively.

We compared our road data with those from the two global-road databases, GRIP and OSM, using the same 1.42 million plots for all datasets. Road extent (the percentage of mapped 1-km^2^ cells containing at least one road) was 13.2% using GRIP and 18.3% using OSM, but a much higher 32.9% when using our road data (Fig. 1b). In addition, the total length of mapped roads was 3.0–6.6 times greater when using our dataset (1.37 million km) than when using the GRIP (0.21 million km) and OSM (0.45 million km) datasets. Compared with GRIP and OSM, our data revealed that 35–45% of unmapped roads were in oil-palm or other plantations (23–33% in large plantations; 11–12% in small plantations), 31–39% were in intact forests and 17–28% were in non-plantation agriculture (see Supplementary Fig. 2 and Supplementary Information for land-use definitions). Unmapped roads were less prevalent in urban areas, degraded forest and other land-use types (Supplementary Fig. 2).

Our findings show that the extent and length of roads, at least in our study area, are severely underestimated in leading road databases and official government statistics (Fig. 1 and Supplementary Table 2). Moreover, these badly deficient road data partly underlie popular conservation metrics, such as the ‘human footprint’ index^21,22^ and ‘roadless’ or ‘wilderness’ areas^5,23^, that are widely used in conservation research and management (see below).

## Modelling forest loss

Next, we tested the relative importance of roads and other potential spatial predictors in driving forest loss in our 1.42 million plots. To do this we first created a comprehensive land-cover map for our study region and then quantified the percentage of land cleared per plot (hereafter termed ‘forest loss’) as our response variable. Our map was explicitly designed to accurately detect forest loss while not misclassifying current land covers, such as oil-palm or wood-pulp plantations, as forested land, or open vegetation, such as wetlands, as deforested land (Supplementary Information). We then identified 38 key environmental, demographic or socioeconomic variables potentially related to deforestation (Supplementary Table 4). Included among these were neighbourhood road density (total length of roads within a 5-km radius of each plot) and road proximity (linear distance of the plot to the nearest road). Much road building in the tropics is linked to agriculture—the largest ultimate driver of deforestation in the Asia-Pacific region^24–26^—which itself is influenced by underlying socioeconomic and demographic factors^27,28^. Roads also promote deforestation by markedly reducing the costs of transporting timber, bulk minerals, fossil fuels and poached wildlife to domestic or international markets^27,29^.

**Fig. 2:**
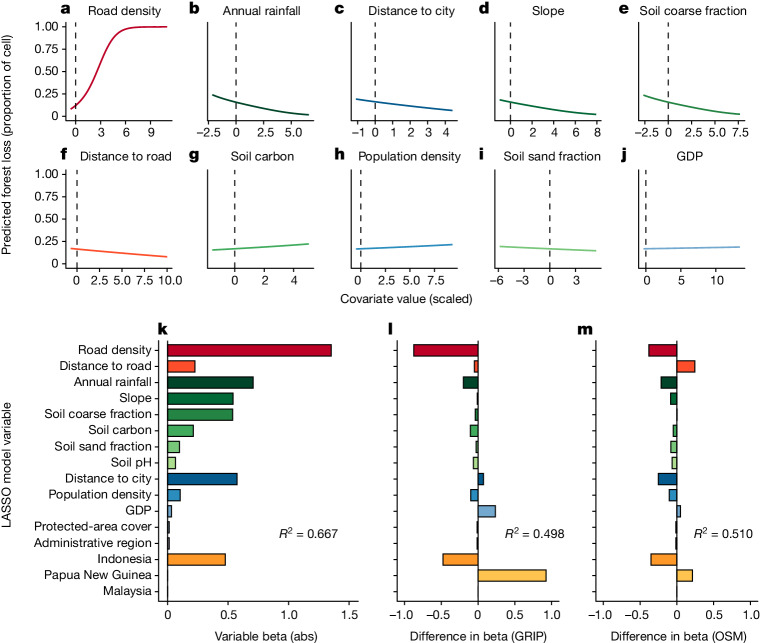
Environmental and socioeconomic features that influence forest loss across the tropical Asia-Pacific region. **a**–**j**, Partial differential plots showing relationships between the 10 most influential features and forest cover (road density (**a**), annual rainfall (**b**), distance to city (**c**), slope (**d**), soil coarse fraction (**e**), distance to road (**f**), soil carbon (**g**), population density (**h**), soil sand fraction (**i**) and gross domestic product (GDP) (**j**)). The *x*-axis values indicate the number of standard deviations from the mean; see Supplementary Table 5. **k**–**m**, Spatial predictors of deforestation, showing slope (beta) values for model using our road data (**k**) and the difference in slope values when using alternative road data from GRIP (**l**) and OSM (**m**). Abs, absolute values.

To model forest loss on the basis of our 38 potential predictor variables, we developed a generalized linear model with LASSO regularization^11^ (a technique that encourages simple, sparse models, with fewer parameters and less model variance and bias). Out of these 38 potential predictors, 14 had a discernible relationship with forest loss (Supplementary Information), and their effects were then contrasted using road datasets from this study, GRIP and OSM (Fig. 2). Notably, the marginal relationship between road density and forest loss was distinctly nonlinear (Fig. 2a). This sigmoidal curve suggests a general threshold effect of roads, with deforestation rates being highest when new roads are first constructed in a landscape, and then gradually decreasing as road density increases. Forests are expected to decline most sharply when roads initially encroach, up to a road density of around 4 km km^−2^, with accessible forests becoming largely depleted if road density exceeds around 7.5 km km^−2^. Broadly similar dynamics have been observed in rural communities experiencing ‘boom-and-bust’ development in the Brazilian Amazon^30^, where initial road building triggers rapid forest loss followed by declines in environmental and human welfare as forest resources are increasingly exhausted.

In our final LASSO regression model, several other variables—annual rainfall, distance to nearest city, topographic slope, soil coarse fraction, distance to nearest road, and country—had modest explanatory power, with each having beta (slope) values significantly smaller than that of road density (Fig. 2b–j). Marginal relationships of these variables with forest loss largely followed expected trends (that is, forest loss was highest near townships or cities, in flatter areas and in less-rainy locales where forest burning is easier) (Fig. 2b–j). Notably, protected-area coverage (with a beta value of just −0.01) had little influence on model performance. We did not evaluate various other potential drivers of deforestation, especially ultimate factors (for example, poverty, access to global markets and social norms)^27^ for which we lacked adequately spatially resolved data. Thus, although road density was the strongest spatial predictor of forest loss in our study, we were unable to consider every conceivable driver of deforestation in our model.

We also ran separate LASSO models for each country and then compared their performance with that of our region-wide LASSO model, which indicated that Indonesia had a higher marginal rate of forest loss than did either Malaysia or Papua New Guinea. Notably, the region-wide model performed better (pseudo *R*^2^ = 0.667) than the three country-level models (pseudo *R*^2^ = 0.540, generated by using area-weighted averages for each nation) (Supplementary Fig. 3). In addition, we reran our LASSO regression while excluding large-scale oil-palm and pulpwood plantations (Supplementary Fig. 4), which are associated with considerable deforestation in the Asia-Pacific region^24^. This produced only negligible changes in model slope parameters and overall outcome (Supplementary Information), underscoring the robustness of our region-wide model.

The LASSO model based on our road data, which included ghost roads, differed in three important ways from those based on the GRIP and OSM datasets (Fig. 2k–m). First, the model with our improved road data was considerably stronger, explaining more of the total deviance in the response variable (66.7%) than did either the GRIP- or the OSM-based models (49.8% and 51.0%, respectively). As a result, our model was better at predicting spatial patterns of forest loss across our study area (Fig. 2). Second, when using our road data, road density was a much stronger correlate of forest loss (with a beta value of 1.35, which is around 1.4–2.8 times greater than OSM- and GRIP-based values, respectively). Third, the effect of country on forest-conversion rates differed substantially (particularly for Indonesia and Papua New Guinea) when using GRIP or OSM data, compared with our comprehensive road dataset. Hence, the widely used GRIP and OSM datasets are not just seriously incomplete but also markedly inconsistent among nations or geographic regions (Supplementary Table 2)—with developing nations generally having much poorer road data than do wealthier nations^5,18^.

## Roads and protected areas

Next, we assessed the degree to which areas that are designated as protected by the International Union for Conservation of Nature (categories I–VI) limit road incursions and forest loss, relative to non-protected areas, using the three road datasets. We first used propensity-score matching^31^ to account for non-random locations of protected areas, such as biases toward steeper or less productive lands (Supplementary Information). We then used separate propensity-score analyses to assess the capacity of protected areas to reduce both road incursions and forest loss.

**Fig. 3:**
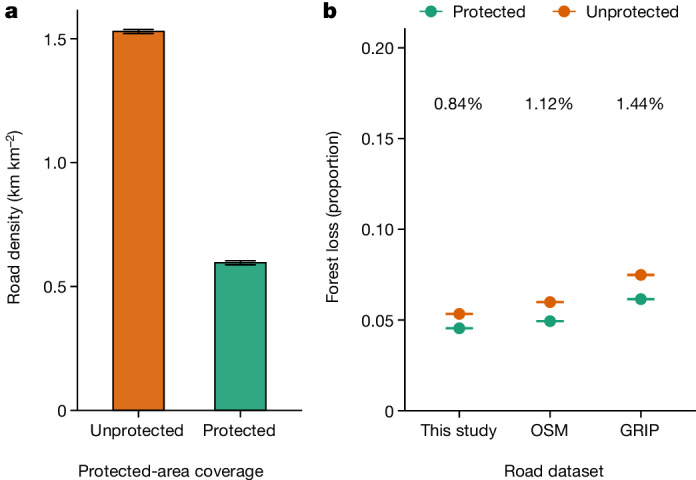
Effects of protected areas in limiting road construction and forest loss. **a**, Differences in road density between protected and unprotected areas after site-matching analysis. **b**, Marginal difference in forest loss between protected and unprotected cells after site matching with the full dataset from this study. For both panels, error bars show 95% confidence limits (in **b**, the error is too small for the gap between bars to be visible).

When comparing matched sites, we found that average road density was more than twice as high (256.7%) outside protected areas than inside them (Fig. 3a). However, after accounting for lower road densities inside protected areas, the marginal effects of protected-area coverage on forest loss were surprisingly modest: less than 1% in magnitude when based on the road datasets from this study, and less than 1.5% in magnitude when based on data from OSM or GRIP (Fig. 3b). This suggests that, on a per-kilometre basis, roads inside protected areas lead to nearly as much forest loss as do roads outside protected areas. We assert that the most crucial conservation function of terrestrial protected areas, at least in the Asia-Pacific region, is limiting road incursions and their many associated impacts on forests.

## Roads precede forest loss

**Fig. 4:**
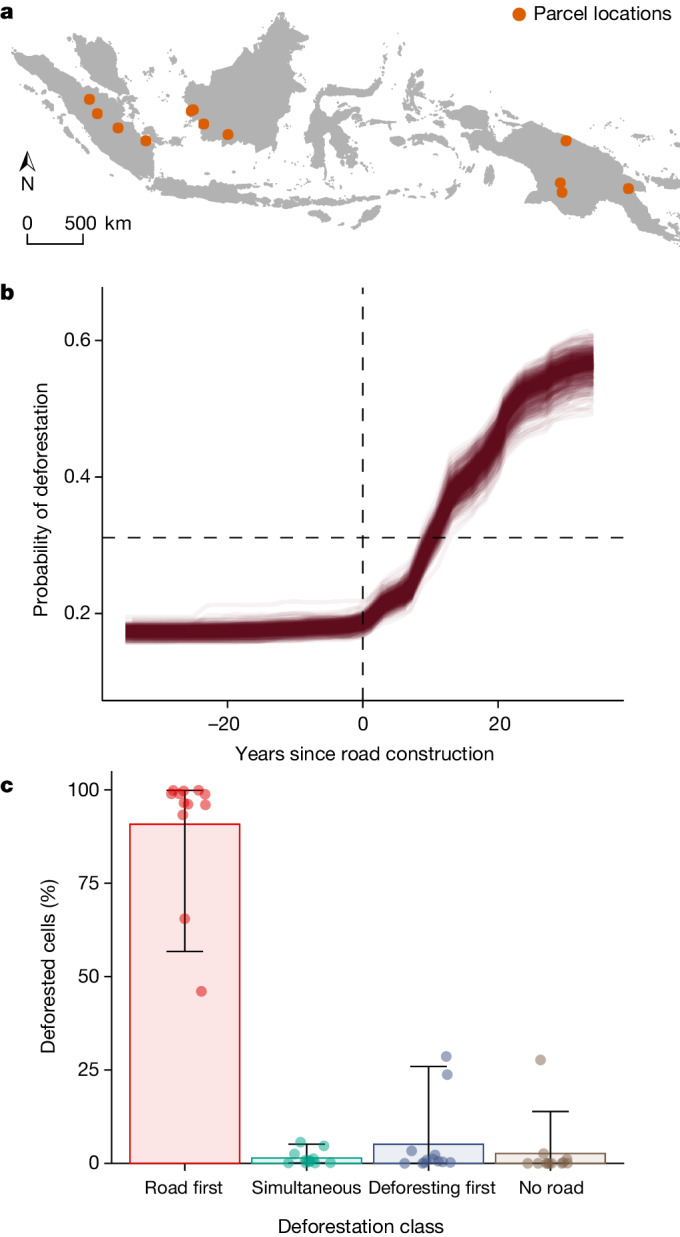
Roads usually precede deforestation. **a**–**c**, Temporal relationships between road construction and deforestation for 12 study sites arrayed across the continental islands of Sumatra, Borneo and New Guinea. **a**, Locations of the 12 study sites. **b**, Partial plots for random forest models testing the temporal relationship between nearby road construction and deforestation (negative values for ‘Years since road construction’ indicate the number of years before road building, whereas the horizontal dotted line shows where true positive and true negative rates are maximized—where cells are more likely to be deforested than not). Each individual line shows the partial plot from a single model iteration. **c**, Median deforestation rates associated with different road-proximity categories. Error bars show the 5–95% interpercentile range for each category.

Finally, to test whether roads tend to precede deforestation, or rather, follow it, we evaluated the temporal sequence of land-use change in 12 large land parcels (each around 400 km^2^ in area) arrayed across Sumatra, Borneo and New Guinea (Fig. 4a). We created 35 annual road maps using annual Landsat imagery from 1985 to 2020 and then identified the spatio-temporal relationship between road construction and deforestation using published annual deforestation data^32^ (Supplementary Information). We summarized this relationship by classifying areas in each parcel that were deforested before, during or after road construction, as well as areas that were deforested independently of roads (more than 2 km from the nearest road).

In our 12 study locations, the probability of deforestation was low before road construction, but spiked immediately after nearby roads were created (Fig. 4b). Our assessment showed that the large majority of deforestation—92.2%, on average—occurred after, or concurrently with, the construction of nearby roads (Fig. 4c). Forest loss preceded road construction in just 5.1% of the total area sampled. These trends indicate that forest loss in our study region is overwhelmingly triggered by ongoing road expansion, rather than vice versa. The 12 study locations include some large-scale oil-palm and pulpwood plantations, in which forest loss also typically followed road construction (Supplementary Fig. 3).

## Summary and conclusions

Using road data generated by trained volunteers, we recorded 3.0–6.6 times more roads in the Asia-Pacific region than were found in leading global-road datasets, while revealing vast numbers of unmapped ‘ghost roads’. These findings have key implications for forest conservation. As a consequence of rapidly proliferating ghost roads, government datasets on roads often have large blind spots and inconsistencies, inhibiting spatial planning, law enforcement and the collection of government rents and royalties on exploited natural resources (Supplementary Table 2).

Striking gaps in road maps are not at all unusual, especially for developing nations^2–10^. For instance, studies in the Brazilian Amazon^6,33,34^, Cameroon^35^ and the Solomon Islands^10,36^ also detected many unmapped or illegal roads, ranging from 2.8 to 9.9 times those recorded in OSM or government sources—values that broadly overlap with and even exceed those observed in our Asia-Pacific study area. Protected areas in this region provided considerable protection against road incursions, containing just a third as many roads as did comparable unprotected areas (Fig. 3a). On a per-kilometre basis, however, roads inside protected areas caused nearly as much forest loss as did those in unprotected areas (Fig. 3b). This underscores, in our view, an urgent need to limit unregulated road expansion in protected areas as a general conservation strategy^37–40^.

**Fig. 5:**
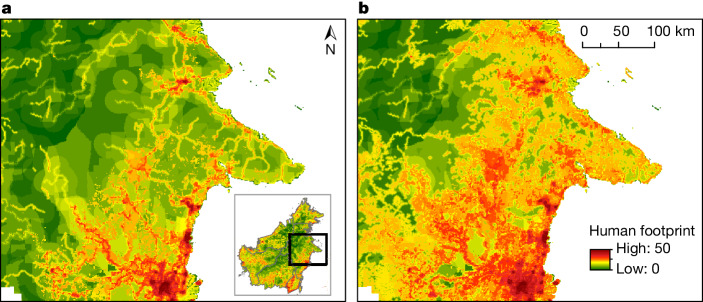
Two versions of the human footprint for eastern and central Borneo, using data from 2020. **a**,**b**, These maps are based on incomplete road data from OpenStreetMap^22^ (**a**) and more-complete road data from this study (**b**).

Although global-road databases are gradually improving in quality^41^, their many gaps and inconsistencies greatly limit their value for comparing different nations, regions and ecosystem types. Furthermore, popular conservation metrics, such as the human footprint^21,22^ and roadless or wilderness areas^23^, are being based in part on seriously incomplete road data. For example, the estimated human footprint in the environmentally critical region of east–central Borneo differs markedly when it is based on a recent OSM road map (Fig. 5a), compared with when it is based on our road data (Fig. 5b). Among these differences, the mapped region in Borneo had twice as much land area with ‘very high’ disturbance (28.4% versus 14.5%), and only half as much land with ‘low’ disturbance (6.6% versus 13.6%), when based on our updated road map and forest-disturbance classifications from the human-footprint study^22^.

The road-mapping element of this study required around 7,000 h of effort by more than 200 trained volunteers or study authors. Such an intensive undertaking is justified only because human eyes still outperform articial intelligence (AI)-based methods for identifying and mapping roads (especially when more-accurate, higher-resolution images are used, as in this study). At larger spatial scales, the required effort is even more daunting. For example, a global-scale analysis using our methods would require around 640,000 h of effort simply to map all of Earth’s current roads just once. For this reason, a viable AI-based road-mapping system is urgently needed^42^. Such schemes are under development^33,43–45^ and could potentially be trained using major datasets such as ours, aiming to provide accurate, global-scale road coverage in near real time. In practical terms, such an automated system is one of the most urgent conservation needs for tropical forests today. Nothing else will keep pace with the contemporary avalanche of proliferating roads.

### Reporting summary

Further information on research design is available in the Nature Portfolio Reporting Summary linked to this article.

## Online content

Any methods, additional references, Nature Portfolio reporting summaries, source data, extended data, supplementary information, acknowledgements, peer review information; details of author contributions and competing interests; and statements of data and code availability are available at 10.1038/s41586-024-07303-5.

### Supplementary information

Supplementary Information Supplementary Text, Supplementary Figs. 1–6, Supplementary Tables 1–5 and Supplementary references.

Reporting Summary

## Data Availability

The datasets used for this study (including comprehensive road maps as a raster of road density at 1-km^2^ resolution) are available in the Supplementary Information, on request to J.E.E. and W.F.L. or as follows. OpenStreetMap data are available from https://download.geofabrik.de/, and GRIP road data from https://www.globio.info/download-grip-dataset. National and subnational administrative-region data were obtained from GADM (https://gadm.org/). Population-density data were from WorldPop (https://www.worldpop.org/). GDP data were accessed at https://datadryad.org/stash/dataset/doi:10.5061/dryad.dk1j0. Protected-area data were from Protected Planet (https://www.protectedplanet.net/en). Waterways locations were obtained from the Global River Widths from Landsat Database (https://zenodo.org/records/1297434). Elevation data were accessed at http://srtm.csi.cgiar.org, and rainfall data at 10.16904/envidat.211. Data for all soil variables were obtained from Soil Grids (https://soilgrids.org/).
