# Supplementary material for: Ghost roads and the destruction of Asia-Pacific tropical forests
Source: Nature. 2024 Apr 10;629(8011):370–5. doi: 10.1038/s41586-024-07303-5 (PMC11078755; doi:10.1038/s41586-024-07303-5)
Supplement: Supplementary file 1 — Supplementary Text, Supplementary Figs. 1–6, Supplementary Tables 1–5 and Supplementary references. [file 41586_2024_7303_MOESM1_ESM.pdf]

nature portfolio
https://doi.org/10.1038/s41586-024-07303-5

Supplementary information

Ghost roads and the destruction of Asia-Pacific tropical forests

In the format provided by the authors and unedited

Table of Contents: Supplementary Information

Page	Description
1	Study region
1	Figure S1: Study area map
1	Comprehensive-roadmap methods
2	Table S1: Sources of road data
2	Table S2: Previously estimated road lengths
3	Methods for comprehensive land-cover map
3	Table S3: Spatial datasets used in analysis
4	Figure S2: Unmapped roads by landcover class
4	Landscape correlates of deforestation
5	Table S4: Potential predictors of forest loss
7	Table S5: Descriptive statistics for modelled variables
8	Figure S3: Results for LASSO-regression models
9	Figure S4: Slope values for LASSO models
9	Effects of protected areas on deforestation
10	Figure S5: Propensity-matching scores
10	Do roads lead to deforestation?
12	Figure S6: Deforestation rates by land-use classes
13	References

Supplementary Information

Jayden Engert *et al.*, Ghost roads and the destruction of Asia-Pacific tropical forests. *Nature* (2024).

Study region

Our study examined the impacts of road building on forest cover in parts of Indonesia, Malaysia, and Papua New Guinea (Fig. S1). These countries were selected because each contains a substantial area of high-value tropical forest under imminent threat from road expansion, agriculture, and extractive industries, as revealed by our recent work[46-50]. Within these three countries, we arrayed our 1-km^2 plots to capture the full spectrum of forest conditions—ranging from areas with intact forest cover and sparse human populations, to areas with devastated forests and high human numbers. In terms of land area, our mapping efforts encompassed 51.4% of Sumatra, 65.6% of Borneo, and 100% of New Guinea.

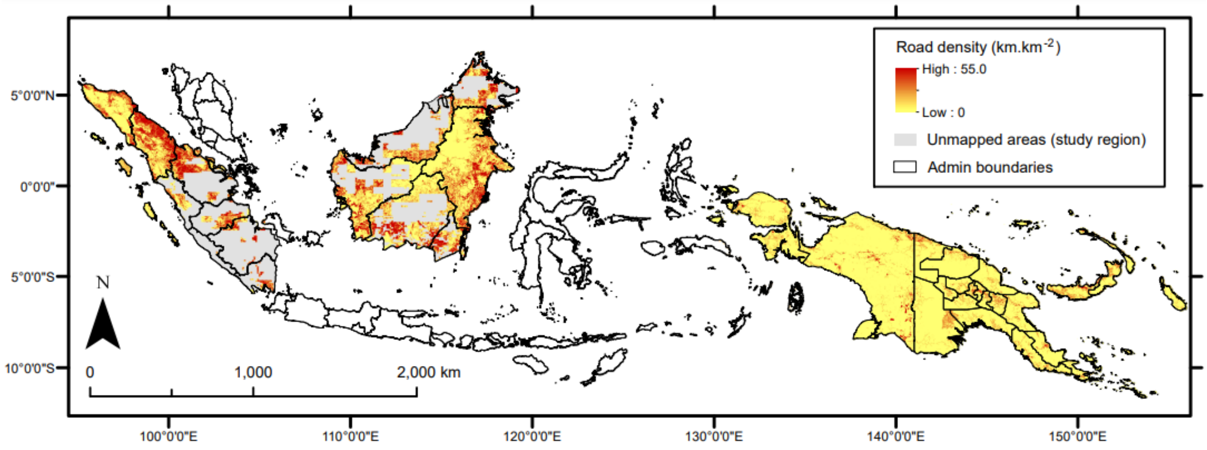

Figure S1. Road density across mapped areas of the study region. The study region consisted of Sumatra, Borneo, and New Guinea, three of the world's largest islands.

Comprehensive roadmap

We gleaned data on existing roads from several sources, including OpenStreetMap and government maps and databases (Table S1). The GRIP dataset was created in 2018 and is not updated, whereas OpenStreetMap is constantly updated with available datasets and citizen-science mapping. We then mapped and digitized all visible roads that were not recorded in these existing road datasets, using Google Earth (circa 2019). Roads were added to new kml files using the New Path tool and by tracing the location of all visible roads in the satellite image, and then exported to shapefiles in Arcmap 10.8. Road-mapping was conducted by 5 coauthors of this study and by 205 trained volunteers, whose individual mapping accuracy was independently quality-checked by one or more study coauthors. Using a standardized protocol that we developed[51], the newly created road files were required to achieve a minimum accuracy of ≥90%, verified using standardized test datasets. Road density was quantified by calculating road lengths at 1-ha grid resolution and then converting this to a raster dataset with 1-km^2 resolution by summing the values.

Table S1. Sources of road data for countries in the study region

Country	Existing road-data source	Reference
Indonesia	Badan Informasi Geospatial	Ref. 52
Papua New Guinea	OpenStreetMap	Ref. 53
	PNG National Mapping Bureau	Ref. 53
Malaysia	OpenStreetMap	Ref. 54
	Borneo Logging Roads	Refs. 55 & 56

We compared our final road dataset to the two most widely used and freely available road datasets (Table S2), the Global Roads Inventory Project (GRIP)[57] from 2018, and a recent version (2020) of OpenStreetMap (https://www.openstreetmap.org). We coined the term ‘ghost roads’ to denote roads that were detected in our comprehensive mapping study yet absent from available sources including GRIP, OSM, and official roadmaps. Official road data for Indonesia were government road maps obtained through Badan Informasi Geospatial[52], whereas official road data for Malaysia were government road maps used to produce the GRIP dataset[57].

Table S2. Estimated length of roads (km) within five broad governance regions in the Asia-Pacific region based on this study and available road datasets. Numbers in parentheses are the proportions of roads across our study region.

Region	This study	GRIP (2018)	OSM (2020)	Official data
Indonesian Borneo	521,617	84,475 (0.16)	136,518 (0.26)	266,591 (0.51)
Indonesian Papua	68,693	20,657 (0.30)	36,643 (0.53)	46,667 (0.68)
Malaysian Borneo	161,395	3,438 (0.02)	45,107 (0.28)	3,438 (0.02)
Sumatra (Indonesia)	522,174	69,948 (0.13)	189,230(0.36)	325,252 (0.62)
Papua New Guinea	97,519	29,618 (0.30)	41,017 (0.42)	-----

Comprehensive land-cover map

Studies on drivers of deforestation often employ simple forest-cover datasets that use remote sensing techniques to classify land based on proportional forest cover. However, these datasets may be limited in their capacity to differentiate between intact forest and tree plantations (such as oil palm or wood-pulp species) or between intact non-forest vegetation types (such as wetlands or grasslands) and cleared land.

To minimize these limitations, we developed a comprehensive land-cover dataset for our Asia-Pacific study area using multiple datasets designed to classify various land-cover types, as well as a general land-cover dataset. These land-cover types were then defined as either ‘intact’ or ‘converted’ land to quantify drivers of human land conversion (Table S3). Land-cover was initially classified at 1-ha resolution, then aggregated to 1-km^2 resolution for analysis by calculating the proportion of 1-ha cells within each 1-km^2 plot that was ‘intact’. Because datasets included in this map are from different time periods and with different temporal resolution, our final composite map does not have any temporal dimension. All cells in which the most common land cover class (by proportion of the plot area) was water were excluded from the model routine.

We used multiple sources[56-63] to estimate the 'original' (pre-clearing) vegetation types within our study region. Overall, forests and forested wetlands (such as peat-swamp forest) were the dominant pre-clearing vegetation types, covering 87.7% of the study area. Other land-cover types, such as non-forested wetlands (8.7%), water bodies (2.8%), and grasslands and other non-forest vegetation (0.8%), were far less abundant[58-65].

Table S3. Spatial datasets used to create the comprehensive land-cover map

Land-cover type	Data description	Order	Class	Reference
Tree plantations	Composite dataset on tree plantations	1	Converted	Ref. 58
	Remotely sensed land-cover map 2020	2	Converted	Ref. 59
	Remotely sensed oil palm plantations	3	Converted	Ref. 60
	Digitized farms and plantations	4	Converted	Ref. 61
Croplands	Remotely sensed cropland extent 2015	5	Converted	Ref. 62
Forest cover	Remotely sensed forest cover 2020	6	Intact	Ref. 63
Rivers	Remotely sensed inland water and rivers	7	Intact	Ref. 64
Intact non-forest vegetation	Remotely sensed land-cover 2019	8	Intact	Ref. 65
All modified land-cover	Remotely sensed land-cover 2019	8	Converted	Ref. 65
Urban areas	Remotely sensed land-cover 2019	8	Converted	Ref. 65
Water	Remotely sensed land-cover 2019	8	Intact	Ref. 65

Ghost-road landcover classes

We used our comprehensive landcover dataset to identify whether ghost roads were predominant in specific land use types [e.g. intact forest, large productive landscapes (>10^4 ha), or small-scale farms]. We used the Region Group tool in Arcmap 10.8 to measure the area of contiguous patches of different landcover types, using a 4-cell neighborhood with the original 1-ha landcover data. Each 1-ha cell therefore was categorized by its landcover type and the size of the discrete patch in which it was included. Using visual inspection of the spatial data, we identified a size-threshold (>10^4 ha) to discriminate large agro-industrial landscapes from other landcover classes. We calculated the length of roads in each 1-ha cell from our dataset for each landcover type, then did the same for the GRIP and OSM datasets separately. To visualise the unmapped roads by each landcover type, we present the difference between our data and the GRIP and OSM as the percentage of total unmapped roads (Fig. S2). Our results show ghost roads tend to occur most commonly in plantations (36% and 45% relative to GRIP and OSM datasets, respectively), but almost as commonly in intact forests (31% and 39% relative to GRIP and OSM datasets, respectively) and to a lesser degree in other agricultural landscapes (28% and 17% relative to GRIP and OSM datasets, respectively).

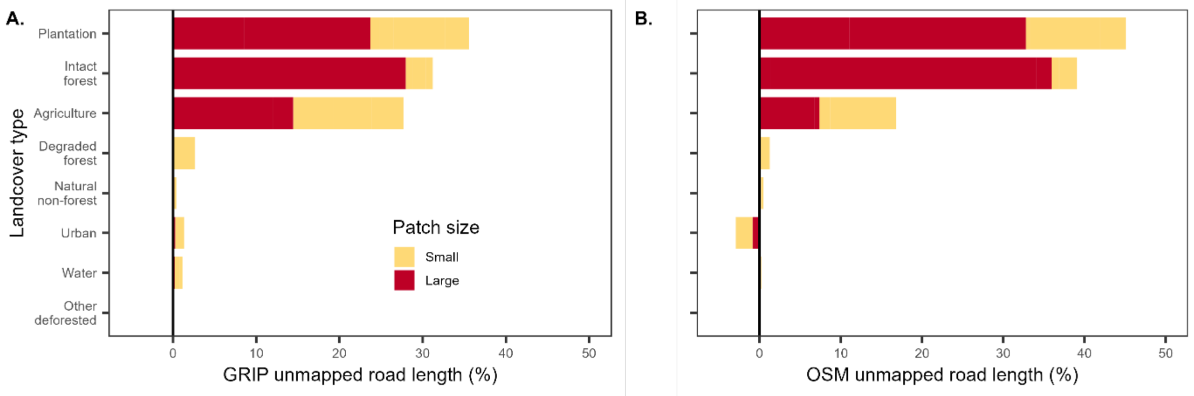

Figure S2. Percentage of unmapped roads by landcover type in the Asia-Pacific region, based on differences between our data and the GRIP and OSM datasets. "Plantation" indicates oil palm, pulpwood, or other tree monocultures, whereas "Agriculture" includes practices such as swidden farming, rice, and coffee production. The frequency of patch sizes is shown separately for smaller (<10,000 ha) and larger (>10,000 ha) plantations.

Landscape correlates of deforestation

Potential spatial predictor variables

A large number of potential drivers of forest loss have been identified in earlier studies[66,67], sometimes operating at widely varying spatial scales[68]. We initially identified 38 socioeconomic and environmental variables (Table S4) that could be related to land conversion and forest loss, particularly via smallholder and industrial agriculture, the largest underlying driver of deforestation in our study region[69,70].

We classified the 38 potential spatial predictors based on whether they represented proximate, underlying, or environmental drivers (Table S4). Proximate drivers are local-scale human activities that directly influence forest loss[66]. In our study, proximate drivers include road density, proximity to roads, and conversely, whether a particular forest tract is located in a protected area (Table S4). Underlying drivers in our study are structural traits in the social system (e.g. human population size, market integration, affluence) that can indirectly affect land use by mediating the proximate drivers[66]. Such underlying drivers include national and sub-national administrative regions (reflecting national and local-scale policies), population density, distance to the nearest settlement, and distance to the nearest city (market) (Table S4). Environmental drivers include elevation, slope, slope position, topographical roughness, rainfall, and soil characteristics (Table S4). Five of these variables were quantified at varying spatial scales (e.g. population density was recorded for each 1-km^2 plot as well as for 5, 10, 20, 50, and 100 km radii around each plot). These data were generated using the Focal Statistics tool, and distance-based variables with the Euclidean Distance tool, in Arcmap 10.8.

Wherever possible, spatial data for the 38 variables were sourced from peer-reviewed global datasets or satellite data with a global extent, to maximize the generality of our models. We strove to use the most accurate spatial data available for our study area, but note that differences in spatial resolution, quality, and precision of different datasets can influence their perceived importance in modelling routines. Most of our variables had minimal

modification from the original source material with many were simply transformed to the Asia South Lambert Conformal Conic projection system (https://epsg.io/102012) and resampled to 1-km spatial resolution, during which all layers were snapped to the same extent for ease of analysis. To extract data for these variables, all 1-km resolution cells within the mapped region were converted to points in ArcGIS 10.8, and data from all raster layers were then extracted using the Extract Multi Values to Points tool in ArcGIS.

Model routine

Because our initial modelling included many potential predictors (n = 38) and large numbers of observations (n = 1,418,755 plots), we utilized generalized linear modelling with LASSO (L1) regularization. LASSO regression uses covariate shrinkage to create parsimonious models in the presence of numerous covariates while avoiding over-fitting, and is also effective at dealing with multi-collinearity and complex relationships[71].

We implemented LASSO regression using the cv.glmnet function in the 'glmnet' package in the *R* program[72]. We initially fitted a LASSO regression using all potential predictors of land conversion (Table S4) and selected variables that were retained for the lambda value at which model deviance was minimized (lambda.min). To further minimize potential collinearity, we removed potential predictors that were quantified at multiple spatial scales, keeping only the most influential (highest absolute beta value). For example, road density was considered influential at both 1-km and 5-km scales, but we retained only the 5-km covariate for further steps as this was more influential. This strategy reduced the number of potential predictors from 38 to 14.

Our response variable, forest loss, was quantified as a proportional value with a relatively high frequency of 0 and 1 values. To accommodate this inflation of 0 and 1 values, we fitted LASSO regressions using a quasi-binomial error distribution. Model fitting was assessed using the plotres function in the 'plotmo' package in R[73]. Influential predictors were chosen using LASSO regression with spatial cross-fold validation. The final model included both spatial cross-fold validation and a spatial autoregressive (SAR) term to account for spatial autocorrelation. Model performance (R^2) was calculated while holding the SAR term at its mean value to negate its influence on model performance.

Table S4. Potential predictors of forest loss used in this study, highlighting our rationale for inclusion, spatial scale, and data sources. Each variable is categorized as a proximate driver (e.g. a socioeconomic process or condition that directly leads to forest loss), an underlying driver (e.g. a process or condition that indirectly affects forest loss), or an environmental driver (e.g. geographical features such as slope and soil features that can affect agricultural viability).

Predictor variable	Spatial scale	Rationale	Type of driver	Data source
Country	1 km	Previous work has shown that governance (defined by administrative regions) has direct and indirect effects on agricultural expansion and associated land conversion[74,75].	Underlying	Ref. 76
Administrative region (subnational)	1 km		Underlying	
Population density	1 km	Previous work has shown that population density is positively	Underlying	Ref. 80
	5 km*		Underlying	

	10 km*	associated with both road density[77] and land conversionn[78,79]	Underlying	
	20 km*		Underlying	
	50 km*		Underlying	
	100 km*		Underlying	
Distance to city (>10 people ha^{-1})	1 km	Agricultural location theory predicts that agricultural uses vary with distance from cities[66,81,82].	Underlying	Ref. 80
Distance to village (>4 people ha^{-1})	1 km		Underlying	
GDP	1 km	Gross Domestic Product (GDP) is a measure of affluence. Resource-frontier theory predicts that affluence (in combination with land accessibility and demographic pressures) encourages rapid agricultural expansion[67]. It is therefore likely to correlate with road density and land-cover change.	Underlying	Ref. 83
	5 km*		Underlying	
	10 km*		Underlying	
	20 km*		Underlying	
	50 km*		Underlying	
	100 km*		Underlying	
Protected area coverage	1 km	Previous work has shown that protected areas reduce the amount of land conversion associated with roads[78,84].	Proximate	Ref. 85
Road density	1 km	Numerous studies have noted the effects of roads on land conversion[67,84]; hence, we expect that greater amounts of roads in a given area would produce greater rates of land conversion.	Proximate	Created from data produced by the authors
	3 km*		Proximate	
	5 km*		Proximate	
Distance to road	1 km	Previous work has shown that distance to road has a substantial effect on land conversion rates[78,84].	Proximate	Generated using the Euclidean distance tool in ArcMap 10.8.
Distance to river	1 km	Previous work has shown that distance to navigable rivers can correlate with deforestation rates[84].	Proximate	Generated using the Euclidean distance tool in ArcMap 10.8.
Elevation		Higher elevations often have lower deforestation rates due to inaccessibility [55].	Environmental	Ref. 87
Slope	1 km	Slope has been shown to have a negative effect on land conversion[55].	Environmental	Ref. 87
Slope position	1 km	Slope position is hypothesized to influence land conversion as it affects the accessibility of land.	Environmental	Created using the Geomorphometry and Gradient Metrics Toolbox[88].
Topographical roughness	1 km	Topographical roughness is an alternative classification of topography based on neighboring slope values and is hypothesized to influence land conversion in a similar way to slope.	Environmental	Created using the Geomorphometry and Gradient Metrics Toolbox[88].
	5 km*		Environmental	
	10 km*		Environmental	
	20 km*		Environmental	
Mean annual rainfall	1 km	Rainfall is hypothesised to influence road density and land conversion as high rainfall rates are detrimental to road construction and durability in both low-slope and high-slope regions[49] and impede road use.	Environmental	Ref. 89
	5 km*		Environmental	
	10 km*		Environmental	

Soil bulk density	1 km	Soil characteristics influence land suitability for productive land uses[90] as well as suitability for road and building construction[91].	Environmental	Ref. 92
Soil clay fraction	1 km		Environmental	
Soil sand fraction	1 km		Environmental	
Soil pH	1 km		Environmental	
Soil cation exchange	1 km		Environmental	
Soil C content	1 km		Environmental	

*Diameter of focal neighborhood across which values were calculated.

Spatial autocorrelation

Spatially structured data, such as those in this study, are subject to spatial autocorrelation. Presence of spatial autocorrelation in model residuals violates the assumption of independence and can inflate the Type I error rate[93], potentially leading to selection of unimportant explanatory variables and poorly estimated model parameters[94]. We opted to manage spatial autocorrelation with an autoregressive approach[95,96], as it is less likely than other methods to bias model-parameter estimates. To create the most parsimonious LASSO model while accounting for spatial structure in our data, we used 18-fold spatial cross-validation[97,98].

To determine if we had adequately accounted for spatial autocorrelation in our analysis, we calculated Moran's Index (I), which measures the strength of the correlation between observations dependent on their geographical distance[99]. Due to computational limitations, Moran's I was calculated for model residuals at each spatial fold using the moranfast package in R[100], and the values averaged to estimate the global Moran's I. Moran's I values for our LASSO regressions and ghost-roads dataset, as well as those for the OSM and GRIP data, were all close to 0, indicating a negligible effect of spatial autocorrelation in our models (I values = 0.05±0.02, 0.08±0.04, and 0.08±0.03, respectively).

Table S5. Mean and standard deviations for selected modelled variables (see Figure 2)

Model variable	Mean	SD	Units
Road density (5-km radius)	1.55	2.59	$km \cdot km^{-2}$
Topographic slope	8.36	8.41	percent
Soil coarse fraction	81.04	30.49	$cm^{3} \cdot m^{-3}$
Distance to nearest city	87.14	79.56	km
Distance to nearest road	49.23	70.38	km
Soil organic carbon content	991.1	646.5	$g \cdot kg^{-1}$
Annual rainfall	3,112.1	872.8	mm
Soil pH	5.07	0.44	pH
Population density (100-km radius)	1,136.8	2,308.0	People$\cdot$ km^{-2}
Soil sand fraction	297.0	50.9	g·kg
GDP (100-km radius)	593.0	1,680.0	Million US$\$ \cdot km^{-2}$

Country-level deforestation models

The three countries in this analysis have different road densities and rates of deforestation, and we therefore tested whether country-specific LASSO models outperformed a single region-wide model. Our region-wide model performed better than the country-level models for both Indonesia and Papua New Guinea, and performed better overall for the whole region.

Conversely, the country-level model for Malaysia performed better than did the region-wide model for Malaysia (Fig. S3). For all three models, road-related variables had the greatest effect on forest loss. Although Papua New Guinea had the lowest road density, the beta (slope) terms were similar for both PNG and Indonesia, suggesting that roads had comparable effects on forest loss in both regions. Despite minor differences among countries, the region-wide model still had the best overall performance, and thus we used that model for the subsequent analyses.

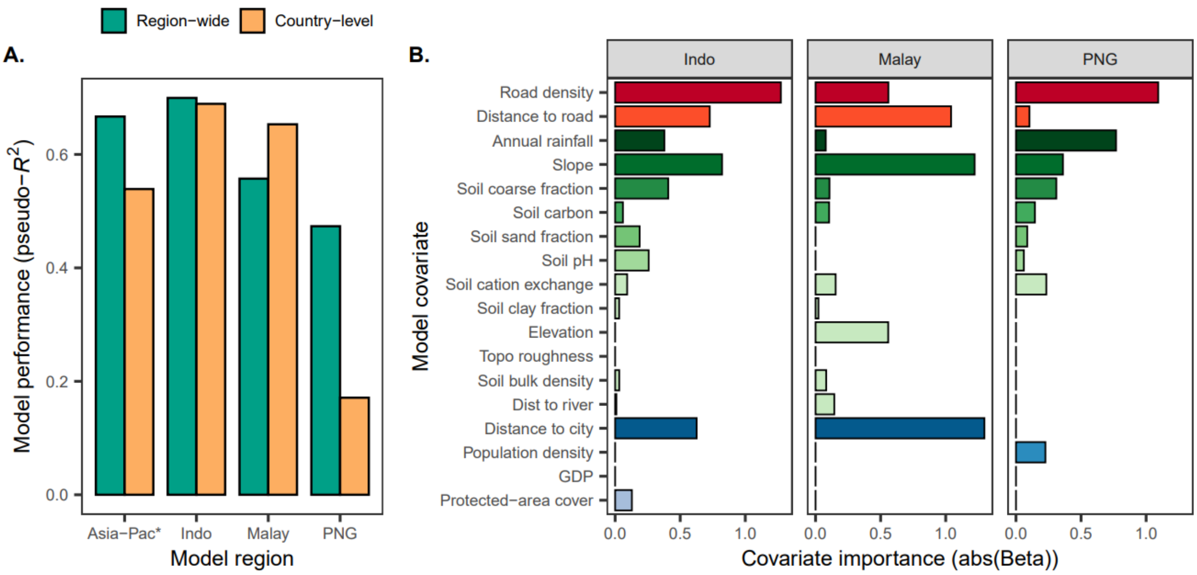

Figure S3. Model performance and variable importance for a region-wide LASSO regression model and for three country-specific models. The pseudo-R^2 value reported for the Asia-Pacific region is the area-weighted mean of the three country-level models.

Model sensitivity to large-scale plantation landscapes

As with the spatio-temporal analysis, we assessed whether large-scale plantation landscapes affected the results of our LASSO regression by re-running the model but excluding large plantations. There was almost no difference in model performance or covariate importance when larger plantations (those larger than 10,000 ha, or those larger than 1,000 ha) were each excluded in turn (Fig. S4). Hence, we used the complete dataset, including large plantations, for our deforestation models (Fig. 2).

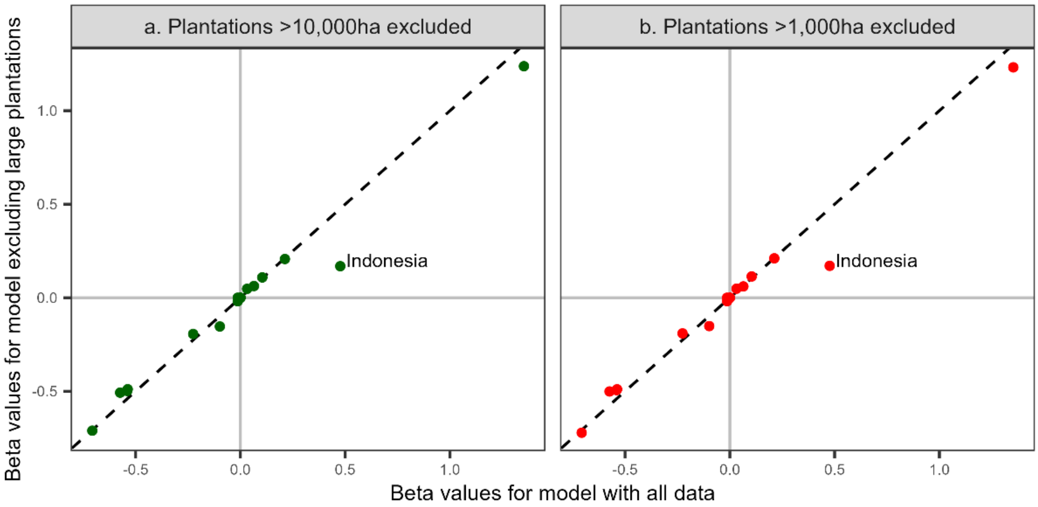

Figure S4. Beta (slope) values for covariates in a LASSO-regression model based on our entire Asia-Pacific-region dataset, versus the same model excluding large-scale plantation landscapes. (a) Comparison of beta values when plantation landscapes larger than 10,000 ha were excluded. (b) Comparison of beta values when plantation landscapes larger than 1,000 ha were excluded. Indonesia (labelled) was the only model term in which the beta value was noticeably altered when large plantations excluded.

Effects of protected areas

We used propensity-score matching[101] to determine whether protected areas[85] limit road incursions relative to non-protected areas, and whether protected areas limit forest disruption when roads are present. This method compensates for potential biases in protected-area location[102] (e.g. the tendency for reserves to be established in steep terrain, where few roads are present). Before matching, all of the 1-km^2 cells in each plot were removed if the dominant land-cover class was water, or if the 1-km^2 cell was partially inside a protected area.

To analyse the effect of protected areas on forest loss, protected and unprotected cells were matched using all influential deforestation drivers identified in our LASSO models (apart from protected-area coverage). Site-matching analysis was conducted without replacement using the MatchIt package in R[103], using a 'nearest neighbor' matching method with glm distance and a relatively strict calliper of 0.01 standard deviations. The robustness of site-matching was assessed by comparing the balance of the two datasets (Fig. S5). Following site-matching, we calculated the marginal effect of protected areas on land conversion using a block-bootstrapping method[104].

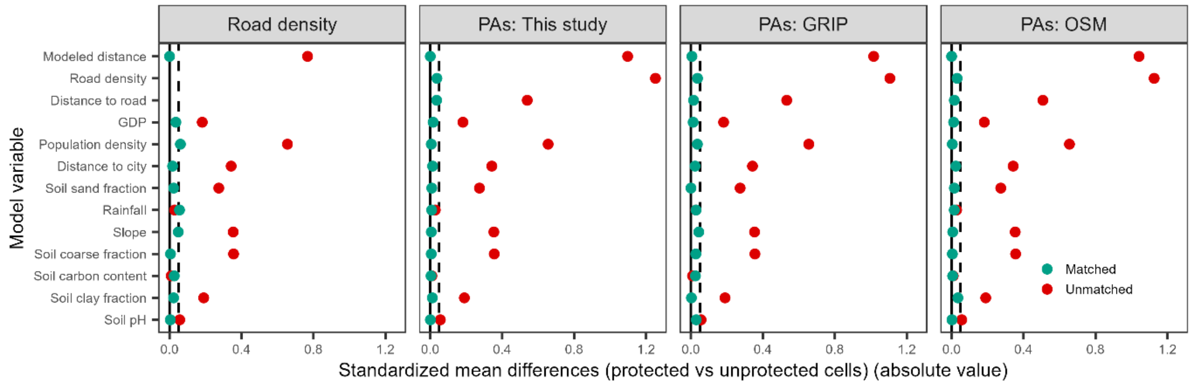

Figure S5. Standardized mean differences between protected and unprotected cells before (red circles) and after (green circles) propensity-score matching. Smaller standardized mean difference values indicate greater similarity between values in protected and unprotected sites. Dashed lines indicate a standardized mean difference of 0.05.

Finally, to assess the effect of protected areas on road incursions (Fig. 3), cells for protected and unprotected areas were matched using the influential forest-loss drivers (excepting variables for road density, distance to nearest road, and protected-area coverage). After site-matching, the marginal effect of protected areas on road density was calculated using the coeftest function in package 'lmtest'[105].

Do roads lead to deforestation?

Our analyses reveal that proliferating roads are strongly associated with forest loss across our study area (Fig. 2). To assess whether roads typically precede, or rather follow, deforestation, we created spatio-temporal maps of road-network expansion. Using Landsat imagery accessed via Google Earth, we created annual road maps for each year from 1985 to 2020 inclusive by mapping all visible roads every year for 12 large parcels (each ~400 km^2 in area) arrayed across our study area. These parcels (Fig. 4) were selected using four criteria: (1) they were arrayed evenly (four plots each) across the three large islands in this study; (2) they occurred in sites that had little if any road construction or forest loss prior to 1990 (because most deforestation in the region has occurred since that date); (3) they broadly sampled prevailing landcover types (small- and large-scale plantations and various agricultural lands) in the region; and (4) each parcel had >50% forest loss by 2020 to ensure it included sizable areas of roads, forests, and deforested lands to evaluate in our analysis. Once mapped, forest roads were retained in the analysis even if no longer detectable, as regeneration of the forest canopy can obscure roads in satellite imagery in just a few years.

For each parcel, we digitized all detectable roads each year between 1985 and 2020, and then used these annual roadmaps to calculate the linear distance to the nearest road for each year at 1-ha raster resolution. We identified the year in which the majority of deforestation occurred for each 1-ha raster cell using the Resample function in Arcmap 10.8 on published deforestation data[106]. We also assumed that all deforestation located <2 km from a road was associated with that road (in our study area, >80% of all deforestation was <2 km from one or more roads) and conducted a sensitivity analysis to ensure this threshold did not influence the results.

We identified the spatio-temporal relationship between road construction and deforestation using random forest models. Random forest models are robust to complex non-linear relationships[107] and suitable for model averaging techniques that reduce computational burden. For each 1 ha cell that was within 2 km of a road, we calculated the number of years since (or until) road construction by identifying the first year the cell was within 2 km of a road and subtracting this value from the current year, and used this term as a model predictor. The model response was a temporal deforestation term that identified if the cell had been deforested in the current year or some previous year (1), or had not been deforested (0).

To account for temporal pseudo-replication, as each cell had 35 observations (1 observation per year), we used the cell ID as a model predictor. In order to reduce computational burden and quantify model uncertainty, we created groups of 1,000 cells and iteratively ran random forest models on each group of cells (circa 411,000 cells that were within 2 km of a road in at least one timestep). We then created partial plots of the relationship between years since road construction and probability of deforestation for visual representation (Fig. 2A) and assessed model performance by predicting from each model and calculating AUC values. Model performance was high with a mean AUC = 0.940. Random forest models were run using the ranger package[108] in R (version 4.1.2), partial plots were created using pdp[109], and AUC values calculated using dismo[110].

Finally, we created a simplified spatio-temporal assessment by classifying each individual 1 ha cell that had been deforested based on the time of deforestation and time of road construction. Cells were classified as either: (1) deforested before road construction (deforest first), (2) deforested concurrent with road construction (simultaneous), (3) deforested after road construction (road first), or (4) >2 km from the nearest road (no road); then we calculated the proportion of each parcel in each of the four deforestation categories. Differences in the frequency of these four categories of landscape change (Fig. 2B) were highly significant ($F_{3,42}$=194.4, P<0.0001), with deforestation after road construction being far more prevalent than any other category (P<0.0001). No other pairwise comparisons were significant (P>0.05). In addition, there were no significant differences in categories among the three large islands in this study ($F_{2,42}$=0.00, P=1.00; two-sided, two-way ANOVA with Tukey's post-hoc tests).

Sensitivity of spatio-temporal analysis to distance threshold

To ensure our decision to use a 2-km distance threshold did not influence the results of the spatio-temporal analysis, we also conducted the aforementioned analyses using 1-km and 3-km distance thresholds. While the probability of deforestation occurring before or concurrently with road construction was slightly higher using a 1-km distance threshold (Fig. S6D, S6E), we found no difference between distance thresholds in the proportion of deforested cells in each deforestation class (Fig. S6E) ($F_{2,139}$ = 0.00, P = 1.00; two-way ANOVA, two-sided). We therefore reported the results using a 2-km distance threshold, as this had a lower proportion of deforested cells not associated with roads than did the 1-km threshold.

Sensitivity of spatio-temporal analysis to large-scale plantation landscapes

Large-scale plantations are more common in some of our study islands, such as Sumatra, than others, such as New Guinea. To ensure that the varying prevalence of large-scale plantation landscapes did not bias our spatio-temporal analysis of roads and deforestation (Fig. 4), we

(1) assessed the amount of each landcover class in our study region, and (2) compared the spatio-temporal relationship between roads and deforestation in large-scale plantation landscapes (>10,000 ha) versus all other landscapes (Fig. S6). While the random forest model suggested that deforestation occurred closer to road construction date in large plantation landscapes (Fig. S6B), we found no significant difference in the spatio-temporal relationship of roads and deforestation when comparing areas inside versus outside plantation landscapes ($F_{1,52}$ = 0.45, P = 0.50; two-way ANOVA, two-sided) (Fig. S6C). Therefore, we conclude that the varying occurrence of large plantation landscapes across our study area did not significantly bias the observed relationship between roads and deforestation.

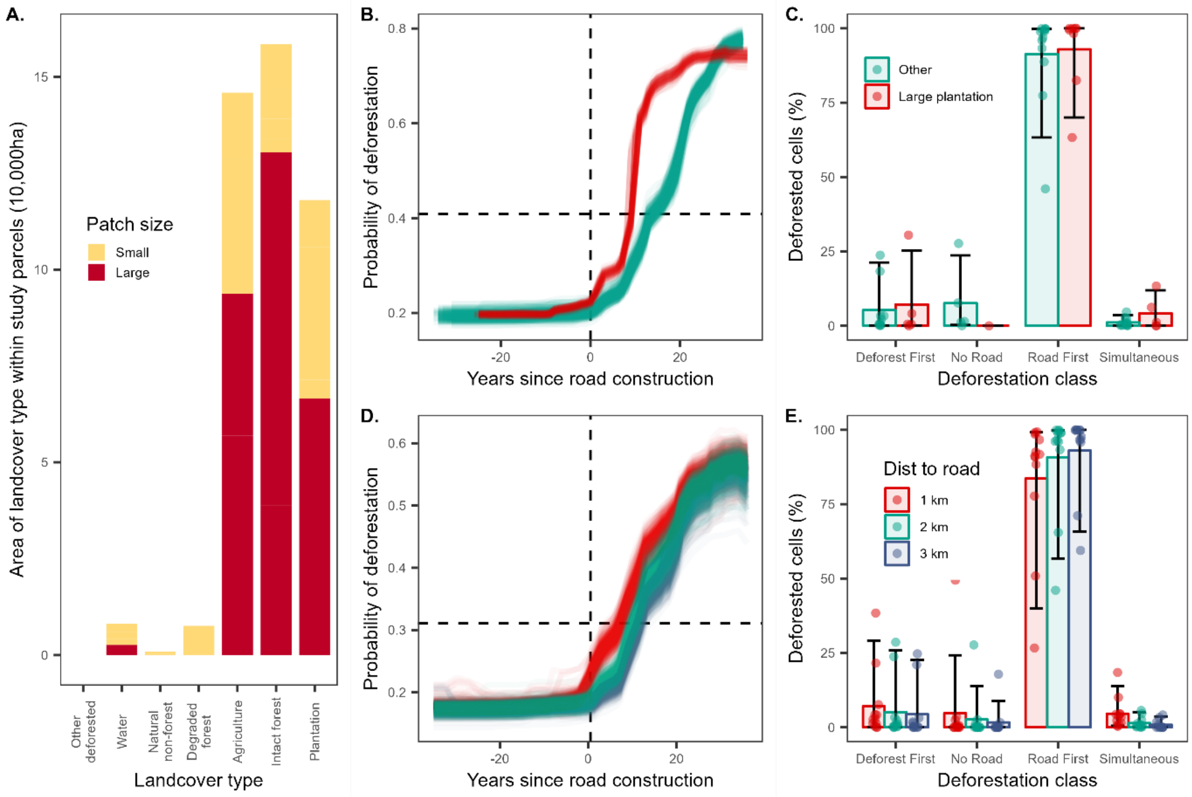

Figure S6. Relationship between roads and deforestation for large agri-industrial lands versus other land-cover types in the Asia-Pacific region and sensitivity of spatiotemporal analysis to distance threshold. (A) The area of each landcover type within the spatio-temporal study parcels. (B) Random forest partial plots of the relationship between years since proximate road construction and the probability of deforestation for areas within large plantation landscapes and all other landcover types. (C) Spatio-temporal relationship between roads and deforestation within large plantation landscapes ("Large plantation") and in all other landcover types ("Other"). (D) Random forest partial plots of the relationship between years since proximate road construction and the probability of deforestation for the three distance thresholds (1 km, 2 km, 3 km). (E) Spatio-temporal relationship between roads and deforestation for three distance thresholds (1, 2, and 3 km). In random forest partial plots, negative values for 'Years since road construction' can be considered 'years until road construction'. For panels (C) and (E), error bars show the 5% to 95% interpercentile range. For panels (B) and (D), each line represents the partial plot from a single model iteration.

References

46. Alamgir, M. *et al.* Infrastructure expansion challenges sustainable development in Papua New Guinea. *PLoS ONE* **14**, e0219408 (2019).
47. Alamgir, M. *et al.* High-risk infrastructure projects pose imminent threats to forests in Indonesian Borneo. *Sci. Rep.* **9**, 140 (2019).
48. Sloan, S. *et al.* Infrastructure development and contested forest governance threaten the Leuser Ecosystem, Indonesia. *Land Use Policy* **77**, 298-309 (2018).
49. Sloan, S. *et al.* Development corridors and remnant-forest conservation in Sumatra, Indonesia. *Trop. Conserv. Sci.* **12**, 1-9 (2019).
50. Sloan, S. *et al.* Trans-national conservation and infrastructure development in the Heart of Borneo. *PLoS ONE* **14**, e0221947 (2019).
51. Sloan, S., Engert, J., Huther, J. & Ishida, Y. *Mapping and Classifying Roads in Insular Southeast Asia Using Google Earth: Orientation Manual* (https://www.global-roadmap.org/wp-content/uploads/2023/02/Mapping_New_Roads_Booklet_v6.pdf; 2020).
52. Badan Informasi Geospatial (2014). Indonesian road maps at 1:50,000 scale (http://tanahair.indonesia.go.id/home/; accessed 7 February 2018).
53. Humanitarian Data Exchange. *Papua New Guinea – Roads.* Humanitarian Data Exchange (https://data.humdata.org/dataset/papua-new-guinea-roads, 2016; accessed 20 June 2019).
54. Haklay, M. & Weber, P. Openstreetmap: User-generated street maps. *IEEE Pervasive Computing* **7**, 12-18 (2008).
55. Gaveau, D.L. *et al.* Reconciling forest conservation and logging in Indonesian Borneo. *PLoS ONE* **8**, e69887 (2013).
56. Gaveau, D.L. *et al.* Four decades of forest loss and degradation in Borneo. *PLoS ONE* **9**, e101654 (2014).
57. Meijer, J.R., Huijbregts, M.A.J., Schotten, K.C.G.J. & Schipper, A.M. Global patterns of current and future road infrastructure. *Environ. Res. Lett.* **13**, 064006 (2018).
58. Harris, N., Goldman, E. & Gibbes, S. *Spatial Database of Planted Trees (SDPT) Version 1.0.* (World Resources Inst., 2019).
59. Lang, N., Schindler, K. & Wegner, J.D. A high resolution canopy height model of the Earth (Cornell Univ., https://arxiv.org/abs/2204.08322, 2022).
60. Descals, A. *et al.* High-resolution global map of smallholder and industrial closedcanopy oil palm plantations. *Earth Syst. Sci. Data*, **13**, 1211-1231 (2021).
61. OpenStreetMap (https://www.openstreetmap.org; accessed 2 October 2021).
62. Thenkabail, P.S. *et al.* Global cropland-extent product at 30-m resolution (GCEP30) derived from Landsat satellite time-series data for the year 2015 using multiple machine-learning algorithms on Google Earth Engine cloud. U.S. Geological Survey Professional Paper 1868, (2021).
63. Hansen, M.C. *et al.* High-resolution global maps of 21st-century forest cover change. *Science* **342**, 850-853 (2013).
64. Allen, G.H. & Pavelsky, T.M. Global extent of rivers and streams. *Science* **361**, 585-588 (2018).
65. ESA. *Land Cover CCI Product User Guide Version 2. Tech. Rep.* (https://maps.elie.ucl.ac.be/CCI/viewer/download/ESACCI-LC-Ph2-PUGv2_2.0.pdf; 2017).

66. Geist, H.J. & Lambin, E.F. Proximate Causes and Underlying Driving Forces of Tropical Deforestation: Tropical forests are disappearing as the result of many pressures, both local and regional, acting in various combinations in different geographical locations. *BioScience* **52**, 143-150 (2002).
67. Meyfroidt, P. et al. Middle-range theories of land system change. *Global Environ Change* **53**, 52-67 (2018).
68. Cushman, S.A., Macdonald, E.A., Landguth, E.L., Malhi, Y., & Macdonald, D.W. Multiple-scale prediction of forest loss risk across Borneo. *Landscape Ecol.* **32**, 1581-1598 (2017).
69. Curtis, P.G., Slay, C., Harris, N., Tyukavina, A. & Hansen, M.C. Classifying drivers of global forest loss. *Science* **361**, 1108-1111 (2018).
70. Pendrill, F. et al. Disentangling the numbers behind agriculture-driven tropical deforestation. *Science* **377**, eabm9267 (2022).
71. Schrieber-Gregory, D. *Regulation Techniques for Multicollinearity: Lasso, Ridge, and Elastic Nets* (https://www.lexjansen.com/wuss/2018/131_Final_Paper_PDF.pdf, 2018).
72. Friedman, J., Hastie, T. & Tibshirani, R. Regularization paths for generalized linear models via coordinate descent. *J. Stat. Softw.* **33**, 1-22 (2010).
73. Milborrow, S. *Plotmo: Plot a Model's Residuals, Response, and Partial Dependence* (https://cran.r-project.org/web/packages/plotmo/plotmo.pdf; 2021).
74. Andersson, K. & Gibson, C.C. Decentralized governance and environmental change: Local institutional moderation of deforestation in Bolivia. *J. Policy Anal. Manage.* **26**, 99-123 (2006).
75. Duran, E., Bray, D.B., Velaquez, A. & Larrazabal, A. Multi-scale forest governance, deforestation, and violence in two regions of Guerrero, Mexico. *World Devel.* **39**, 611-619 (2011).
76. GADM. Database of Global Administrative Areas. Version 4.0 (2021).
77. Glover, D.R. & Simon, J.L. The effect of population density on infrastructure: The case of road building. *Econ. Dev. Cult. Change* **23**, 453-468 (1975).
78. Nzunda, E.F. & Midtgaard, F. Spatial relationship between deforestation and protected areas, accessibility, population density, GDP and other factors in mainland Tanzania. *For., Trees, Livelihoods* **26**, 245-255 (2017).
79. Boserup, E. *The conditions of agricultural growth: The economics of agrarian change under population pressure*. (Routledge, 2014).
80. Tatem, A.J. WorldPop, open data for spatial demography. Scientific data, 4(1), 1-4 (2017).
81. Ahrends, A. *et al.* Predictable waves of sequential forest degradation and biodiversity loss spreading from an African city. *Proc. Nat. Acad. Sci. USA* **107**, 14556-14561 (2010).
82. Thünen, J.H.V., eds. *The Isolated State in its Relation to Agriculture and National Economy* (Oxford: Pergamon, 1966).
83. Kummu, M., Taka, M. & Guillaume, J. Gridded global datasets for Gross Domestic Product and Human Development Index over 1990-2015. *Sci. Data* **5**, 180004 (2018).
84. Barber, C.P., Cochrane, M.A., Souza, C.M. & Laurance, W.F. Roads, deforestation, and the mitigating effect of protected areas in the Amazon. *Biol. Conserv.* **177**, 203-209 (2014).
85. WCMC. *UNEP-WCMC & IUCN World Database on Protected Areas*

(https://www.iucn.org/theme/protected-areas/our-work/world-database-protected-areas; accessed 14 January 2022).
86. Curran, L.M. *et al.* Lowland forest loss in protected areas of Indonesian Borneo. *Science* **303**, 1000-1003 (2004).
87. Jarvis, A., Reuter, H., Nelson, A., & Guevara, E. Hole-filled SRTM for the globe version 3, from the CGIAR-CSI SRTM 90 m database (http://srtm.csi.cgiar.org, 2008).
88. Evans, J.S. & Oakleaf, J. Geomorphometry & gradient metrics toolbox (ArcGIS 10.0, 2012).
89. Karger, D.N., Nobis, M., Normand, S., Graham, C.H. & Zimmermann, N.E. *CHELSATraCE21k: Downscaled Transient Temperature and Precipitation Data Since the Last Glacial Maximum* (https://doi.org/10.16904/envidat.211, 2021).
90. Barrios, E. Soil biota, ecosystem services and land productivity. *Ecol. Econ.* **64**, 269-285 (2007).
91. Lim, S.M., Wijeyesekera, D., Lim, A. & Bakar, I. Critical review of innovative soil road stabilization techniques. *Int. J. Eng.* **3**, 204-211 (2014).
92. Poggio, L. *et al.* SoilGrids 2.0: Producing soil information for the globe with quantified spatial uncertainty. *Soil* **7**, 217-240 (2021).
93. Mets, K.D., Armenteras, D. & Davalos, L. Spatial autocorrelation reduces model precision and predictive power in deforestation analyses. *Ecosphere* **8**, e01824 (2017).
94. Ploton, P. *et al.* Spatial validation reveals poor predictive performance of large-scale ecological mapping models. *Nature Commun.* **11**, 4540 (2020).
95. Dormann C.F. Assessing the validity of autologistic regression. *Ecol. Model.* **207**, 234242 (2007).
96. Crase, B., Liedloff, A.C. & Wintle, B.A. A new method for dealing with residual spatial autocorrelation in species distribution models. *Ecography* **35**, 879-888 (2012).
97. Valavi, R., Elith, J., Lahoz-Monfort, J. & Guillera-Arroita, G. blockCV: An *R* package for generating spatially or environmentally separated folds for k-fold cross-validation of species distribution models. *Methods Ecol. Evol.* **10**, 225-232 (2019).
98. Telford, R. & Birks, H. Evaluation of transfer functions in spatially structured environments. *Quat. Sci. Rev.* **28**, 1309-1316 (2009).
99. Cliff, A. D. & Ord, J. K. Spatial Processes: Models and Applications (Pion, 1981).
100. Cooper, M. Moranfast: Conduct a Quick and Memory-Efficient Moran's I Test. (R Package Version 1.0, 2020).
101. Geldmann, J., Manica, A., Burgess, N.D., Coad, L. & Balmford, A. A global-level assessment of the effectiveness of protected areas at resisting anthropogenic pressures. *Proc. Nat. Acad. Sci. USA* **116**, 23209-23215 (2019).
102. Pressey, R.L. *et al.* Using abiotic data for conservation assessments over extensive regions: Quantitative methods applied across New South Wales, Australia. *Biol. Conserv.* **96**, 55-82 (2000).
103. Ho, D.E., Imai, K., King, G. & Stuart, E.A. MatchIt: Nonparametric preprocessing for parametric causal inference. *J. Stat. Softw.* **42**, 1-28 (2011).
104. Abadie, A. & Spiess, J. Robust post-matching inference. *J. Am. Stat. Assoc.* **117**, 983-995.
105. Hothorn, T. *et al.* Package 'lmtest': Testing linear regression models (https://cran.r-project.org/web/packages/lmtest/lmtest.pdf, 2022).

106. Vancutsem, C. *et al.* Long-term (1990–2019) monitoring of forest cover changes in the humid tropics. *Sci. Adv.* **7**, p.eabe1603 (2021).
107. Breiman, L. (2001). Random forests. Machine learning, 45, 5-32.
108. Wright, M.N. & Ziegler, A. (2017). Ranger: A Fast Implementation of Random Forests for High Dimensional Data in C++ and R. Journal of Statistical Software, 77(1), 1-17.
109. Greenwell, B.M. (2017). pdp: An R Package for Constructing Partial Dependence Plots. The R Journal, 9(1), 421--436.
110. Hijmans, R.J., Phillips, S., Leathwick, J. & Elith, J. (2022). dismo: Species Distribution Modeling. R package version 1.3-9. https://CRAN.R-project.org/package=dismo
